# An Integrated Approach to Identifying Cis-Regulatory Modules in the Human Genome

**DOI:** 10.1371/journal.pone.0005501

**Published:** 2009-05-12

**Authors:** Kyoung-Jae Won, Saurabh Agarwal, Li Shen, Robert Shoemaker, Bing Ren, Wei Wang

**Affiliations:** 1 Department of Chemistry and Biochemistry, University of California San Diego, La Jolla, California, United States of America; 2 Ludwig Institute for Cancer Research and Department of Cellular and Molecular Medicine, University of California San Diego, La Jolla, California, United States of America; University of Glasgow, United Kingdom

## Abstract

In eukaryotic genomes, it is challenging to accurately determine target sites of transcription factors (TFs) by only using sequence information. Previous efforts were made to tackle this task by considering the fact that TF binding sites tend to be more conserved than other functional sites and the binding sites of several TFs are often clustered. Recently, ChIP-chip and ChIP-sequencing experiments have been accumulated to identify TF binding sites as well as survey the chromatin modification patterns at the regulatory elements such as promoters and enhancers. We propose here a hidden Markov model (HMM) to incorporate sequence motif information, TF-DNA interaction data and chromatin modification patterns to precisely identify *cis*-regulatory modules (CRMs). We conducted ChIP-chip experiments on four TFs, CREB, E2F1, MAX, and YY1 in 1% of the human genome. We then trained a hidden Markov model (HMM) to identify the labels of the CRMs by incorporating the sequence motifs recognized by these TFs and the ChIP-chip ratio. Chromatin modification data was used to predict the functional sites and to further remove false positives. Cross-validation showed that our integrated HMM had a performance superior to other existing methods on predicting CRMs. Incorporating histone signature information successfully penalized false prediction and improved the whole performance. The dataset we used and the software are available at http://nash.ucsd.edu/CIS/.

## Introduction

High throughput technologies such as ChIP-Chip [1], [2] and ChIP-sequencing [3], [4] have been successfully applied to map binding locations of individual transcription factors (TFs) at a genomic scale in organisms ranging from yeast to human [2], [5], [6], [7]. Due to the complexity of the human genome and the noise in high throughput measurements, there still exists ambiguity to decide whether the experimental signal reflects the true TF-DNA interaction. In addition, the above technologies only reveal TF binding, which does not necessarily suggest regulatory function of such binding. Given that human genes are often under combinatorial regulation of TFs and the functional binding sites of cooperative transcription factors (TFs) tend to be located close to each other to form clusters in the eukaryotic genome [8], which are often referred as cis-regulatory modules (CRMs), locating CRMs have been proven to be effective on improving the accuracy of predicting TF binding and uncover functional binding sites.

Numerous computational methods have been developed to determine CRMs. Cister [9], COMET [10] and Cluster-Buster [11] use position specific scoring matrices (PSSMs) either known or determined by other means for a pre-selected group of TFs to score genomic regions and find clusters as CRMs. The PSSMs are fixed and not modified during the search for CRMs. In contrast, methods such as CisModule [12] and EmcModule [13] conduct *de novo* identification of CRMs in the sense of simultaneously defining PSSMs for TFs and searching for binding site clusters of these TFs. Additionally, conservation information has also been used to further remove false positives and improve the prediction accuracy [14], [15], [16], [17], [18], [19].

Despite the success of these methods on various cases, there is still much room to improve their performance. Particularly, additional genomic data have been quickly accumulated along with the development of new technologies. Tiling ChIP-Chip array and ChIP-Sequencing technologies provide binding information of TF, which should be informative in predicting CRMs. Recent studies have shown that different regulatory elements such as promoters and enhancers have distinct histone modification patterns [20], [21]. Incorporation of such information into a CRM identification algorithm is also expected to boost its performance.

In this study, we developed a systematic approach to incorporate information of TF binding motif, protein-DNA interaction (ChIP-Chip) and histone modification pattern to locate CRMs. We first conducted TF binding assays using tiling array for four TFs: CREB, E2F1, MAX, and YY1, which often cooperate with one another on regulating gene expression. Limited by the cost of these experiments, the tiling array only covered the ENCODE regions, which is 1% of the human genome.

We present a method to integrate information about sequence, ChIP-Chip experiment and histone modification. Firstly, we refine the PSSMs from TRANSFAC [22] based on the ChIP-Chip ratio using a probabilistic model called GITTAR [23]. The refined PSSMs are used to construct a hidden Markov (HMM) model. To train the HMM we used modified Baum-Welch algorithm to incorporate both sequence and ChIP-Chip ratio. In this configuration sequences are weighted by the ChIP-Chip experiments that binding sequences are boosted and non-binding sites are penalized during the training. To incorporate chromatin modification signatures as additive information, an additional filter is applied to remove predictions that are not supported by the histone evidence.

## Results

### Data Set

We conducted ChIP-Chip experiment using tiling array for 4 TFs: CREB, E2F1, MAX and YY1 in the ENCODE regions (see Methods and Supplementary data). We divided all the probes into positive and negative sets using the *p*-value calculated by an error model: if *p*-value<0.001, positive probe; if *p*-value>0.1, negative probes. The probes with p-values between 0.001 and 0.1 were considered as ambiguous. Because the length of sequence segments generated by sonication is several hundreds of bps, we concatenated nearby positive probes (within 2000 bps) to avoid redundant representation of the same TF binding sites by multiple probes: only the probe with the smallest *p*-value was included in the positive set. In total, we found 373 CREB, 238 E2F1, 962 MAX and 346 YY1 positive probes. 20573, 22501, 19375, and 20723 probes were selected as negatives for CREB, E2F1, MAX, and YY1, respectively (Table 1). The length of probes ranges from 100 bp to 1000 bp. The dataset can be found at http://nash.ucsd.edu/CIS/. Using this dataset we performed five-fold cross-validation tests.

**Table 1:** Dataset of 4 TFs.

	bindings (*p*-value<0.001)	non-bindings (*p*-value>0.1)	ambiguous (0.001<*p*-value<0.1)
CREB	373	20573	3951
E2f1	238	22501	1798
MAX	962	19575	4200
YY1	346	20723	3468

The matrices of the core motif regions for the four TFs are obtained from the TRANSFAC database [22]. We then applied a probabilistic model called GITTAR [23] to further refine the motif matrices and retrieve information beyond the core motif region. GITTAR is a probabilistic model that incorporates sequence motif and ChIP-chip ratio to identify the most reliable binding sites of a TF of interest. The rationale is that the sites of high ChIP-chip ratios, if also containing the binding motif of the TF, are likely to be a true target of the TF. In GITTAR the binding score is calculated
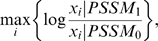
(1)where 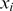 is the *i*th segment of sequence *x* with the core motif in the middle and two flanking regions on both sides. Each segment is selected by allowing 1 mismatches to the core motif. 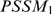 and 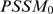 are position specific scoring matrices (PSSMs) for target and background genes, respectively.

**Figure 1:**
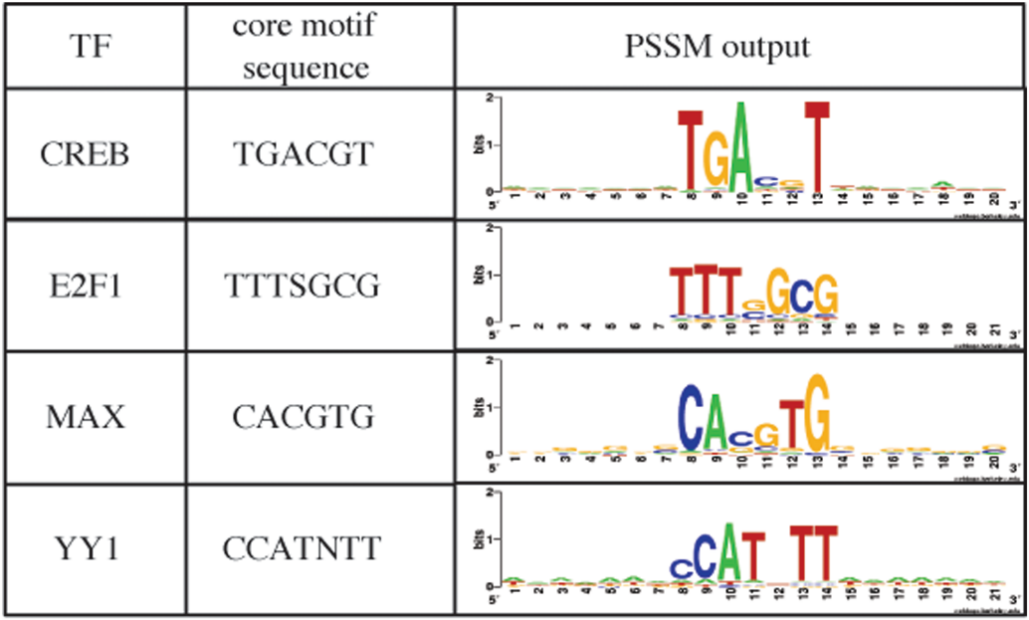
Binding motifs for the four TFs used in establishing the CRM. The sequence logos were generated using WebLogo [30].

As an output GITTAR extended 7 bps at both ends of the core motif and refined the matrix based on the ChIP-Chip ratio. The matrices output from GITTAR (Figure 1) were used in establishing the HMM model for CRM.

### An HMM model for CRM

**Figure 2:**
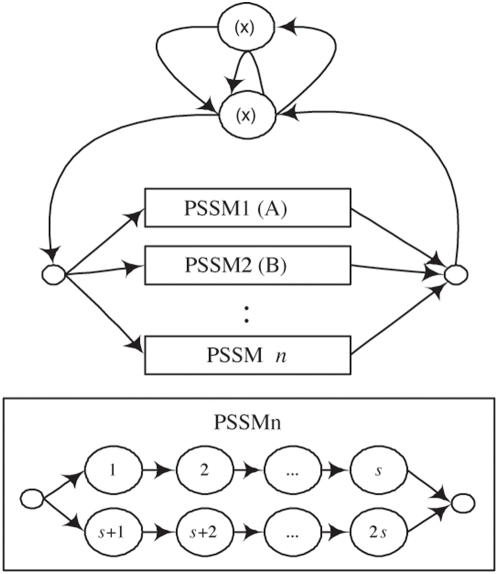
The structure of the HMM with *n* transcription factors (TFs). It is composed of *n* TF blocks and two background states. Between TF blocks and a background block is a branch. Each PSSM block is labeled with an alphabet. Background states are labeled with ‘x’. To model a forward and reverse PSSM, a PSSM block has 2*s*+2 states inside, where *s* is the length of a PSSM.

Previous studies have shown that hidden Markov models (HMMs) [12], [13], [15], [24] are effective in identification of *cis*-regulatory modules. We designed an HMM structure composed of multiple PSSM blocks to train on the DNA sequence as well as the ChIP-Chip ratio(Figure 2). The overall structure of the HMM is similar to those in previous studies such as [10]: there are inter- and intra-module background states to model the regions not bound by TFs and both forward and backward reading of a PSSM are considered in our model.

A unique feature of the current HMM is the labeling of TF blocks. A TF block in the HMM models the binding sites of TFs and both forward and backward strands are considered. Each end of the TF block has a branch used to link the background state to each TF block and does not emit any symbol. The number of the states in a TF block is 2*s*+2, where *s* is the length of a TF matrix obtained by running GITTAR. Associated with each TF block is a label. Including the label for background (‘x’), an HMM with *n* TFs has *n*+1 labels. Each path through the model determines the label of the DNA sequence with the corresponding TFs and the background. This HMM is trained considering sequence information and binding information of a TF to the sequence (see Methods).

Once the HMM is trained, sequences are decoded using posterior algorithm to find a path through the HMM [25]. If a sequence path passes through the labeled states corresponding to a TF, it is regarded as a target of the TF. A sequence can be decoded as a target of multiple TFs if the associated path goes through several labels. The trained model is found at http://nash.ucsd.edu/CIS/.

### Simulation Results

**Figure 3:**
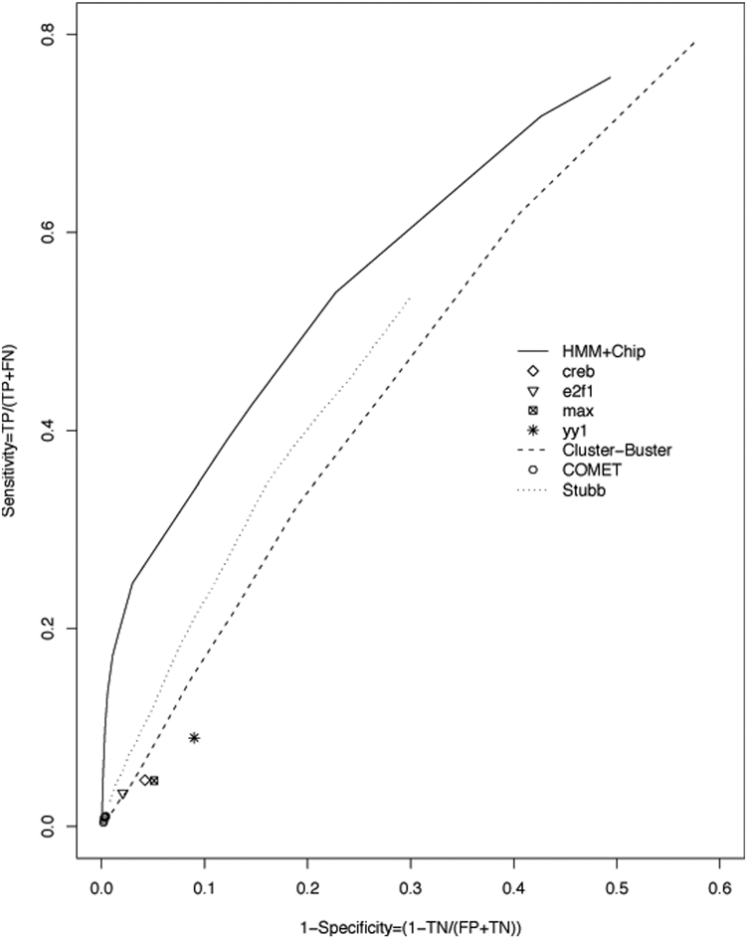
ROC curves for the cis-module predication. The prediction performance of COMET [10], Cluster-Buster [11], Stubb [15] and the proposed HMM approach are compared.

To illustrate the advantages of predicting the CRMs using one model, we compared the prediction accuracy of HMMs that model individual TFs and those that model multiple TFs. The individual and multiple TF HMMs have the same structure and the only difference is the number of PSSM block: one PSSM block in the individual TF HMM and multiple PSSM blocks in the multiple TF HMMs. Individual HMMs for the four TFs were trained using the traditional Baum-Welch algorithm. A prediction is considered as a true positive (TP) if a predicted TF a real target, false positive (FP) if a TF is predicted and the sequence is a non-target, true negative (TN) if a TF is not predicted and the sequence is not a target of the TF, and false negative (FN) if a TF is not predicted while the sequence is a target of the TF. We also defined sensitivity = TP/(TP+FN) and specificity = TN/(TN+FP). Our HMM method using multiple PSSMs was 5 fold cross-validated and the receiver operator characteristic (ROC) curve was generated by increasing the ratio of the background variable (*v_x_*) from 0 until the curve reached the plateau (Figure 3). The proposed method using multiple PSSM blocks showed better performance than the performance over the HMMs using individual HMMs. The success of the proposed model is achieved by combining the 4 TFs and training the combined model while considering experimental binding information of the TFs to a sequence. The individual HMMs showed very good specificity (>0.9) while their sensitivity remained below 0.1. The combined model also showed better performance at the same specificity.

We compared the performance of our method with the existing ones, COMET [10], Cluster-Buster [11] and Stubb [15] (Figure 3). The ROC curves of COMET and Cluster-Buster were generated by increasing the cut-off parameters starting from 0. COMET predicted only 423 targets even using the lowest cut-off (when E-value is 0) and its sensitivity remained below 0.02. Stubb and Cluster-Buster showed better performance than the individual HMMs. Stubb shows superior performance to Cluster-Buster in this test but still worse than the proposed method. To test the usefulness of the evolutionary conservation information we ran Stubb with the aligned human and mouse genomes. This test yielded a result with a very low sensitivity of 0.086.

### Chromatin modification filter

Recent genome-wide surveys have revealed that regulatory elements including promoters and enhancers are associated with characteristic chromatin modification patterns. For example, active promoters are often marked by mono- and tri-methylation of Lys4 in H3 (H3K4Me1 and H3K4Me3); in contrast, much reduced signal of H3K4Me3 are observed for enhancers [21]. Using 10 histone modification markers in the HeLa cell, Won *et al.* developed a computational method to predict promoters and enhancers [26]. We used the 438 promoters and 464 enhancers that Won *et al.* reported in ENCODE region as additional filters to remove false positives of *cis*-module predictions and identify functional sites in the HeLa cell.

The chromatin modification patterns are often spread over thousands of base pairs. We thus used distance from the prediction to promoters or enhancers as a cutoff. We tested the distance ranging from 500 bps to 20 kbps when we applied the chromatin filter and searched for the optimal distance for identifying CRMs. In this configuration only the predictions located within the distance from the promoter or enhancer are only counted. Predictions outside this range were discarded. We defined positive predictive value (PPV) as TP/(TP+FP), negative predictive value (NPV) as TN/(FN+TN) and the Matthews correlation coefficient as 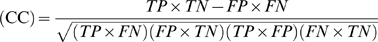 and checked the effect of the histone modification filter.

**Figure 4:**
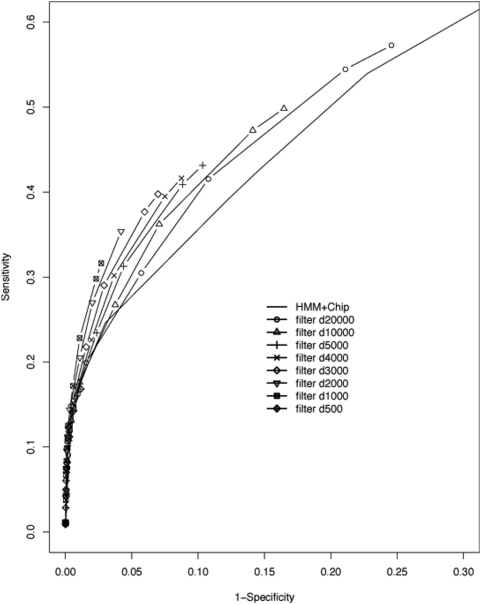
ROC curves after applying the histone modification filter to the predicted CRMs by the HMM. The distance of 20K, 10K, 5K, 3K, 2K, 1K, 0.5K bp (d20000 to d500) to the nearest TSS or p300 binding sites are used in the histone modification filters. A filter with 1∼2 kb distance shows the best performance.

**Figure 5:**
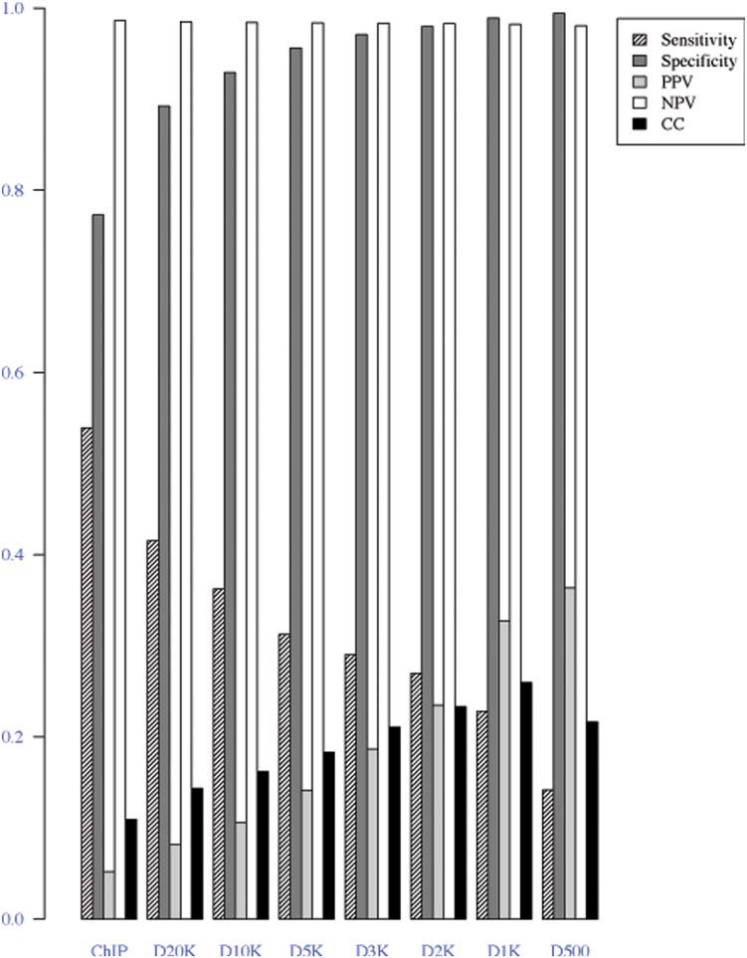
Evaluation of the histone modification filter. Results using various distances (20K, 10K, 5K, 3K, 2K, 1K and 0.5K bp) between the center of the module and that of the predicted promoters from chromatin signature are shown.

Figure 4 shows that the chromatin modification filter restricted false positives and further enhanced the performance of the HMM model. The number of prediction would decrease if we used a more stringent histone modification filter. However, the performance of the predictions was significantly improved: even a loose cutoff (d10000) produced a ROC curve close to an ideal predictor. We observed that the maximum CC was achieved using a distance around 1∼2 Kb. The tradeoff between sensitivity and specificity is further illustrated in Figure 5 and Table 2. When keeping the same number of TPs, we observed dramatic decrease of FPs and increase of TNs by applying histone modification filters. This tradeoff is particularly important for guiding experimental design because often only a limited number of predictions can be tested and a high PPV is desired.

**Table 2:** The prediction performance using different histone modification filters.

	TP	FP	TN	FN	PPV	CC
HMM+ChIP	715	10686	72485	1204	0.06	0.11
D20000	715	7336	75835	1204	0.09	0.15
D10000	715	6437	76735	1204	0.10	0.16
D5000	715	5944	77227	1204	0.11	0.17
D4000	715	5470	77701	1204	0.12	0.18
D3000	715	4841	78330	1204	0.13	0.19
D2000	715	4062	79110	1204	0.15	0.21

TP was kept same.

## Discussion

Sequence analysis has been developed using statistical reasoning based on sequence information and conservation analysis. Though valuable, sequence information does not tell us the whole story about the gene expression. For better prediction performance we introduced ChIP experiment data and histone modification data in constructing our predictor. ChIP-chip data reflects how likely a genomic locus is bound by a TF. When it is included to train the HMM, the genomic regions with low ChIP ratios are penalized in the model. Although ChIP-chip experiments can be quite noisy, including such data is quite useful to weigh sequences in the model training. Because a TF's binding does not necessarily suggest function, additional functional data is obviously useful to further improve the model's performance.

Histone modification data has been shown associated with transcriptional regulation [21] and supplied us with information about biological activity in certain regions. Therefore, it is not surprising to see that histone modification filters can further reduce false positives and increase PPVs. Due to the limit of resources, usually only a handful of predictions can be tested. A high PPV is thus important for guiding experimental validation.

Evolutionarily conserved information has been a useful tool for finding biologically active regions. However, evolutionary information can mislead us in that conservation sequences are also found in many areas besides TFBSs in the genome. Also, recent studies show a rewiring phenomenon that complicates the idea that functionally conserved regulatory regions also share TFBS conservation across species [27]. Histone modification data is quite useful compared to conservation studies because histone modifications strongly correlate with specific biological activities. However, current knowledge about histone modifications is not sufficient to explain the mechanistic role of histone modifications on gene regulation. Moreover, we do not know the long-range effects of histone modifications. In our analysis we found an optimal distance between a modification to a target gene based on optimizing the model's performance. Further evidence is required to better understand the biological reasoning behind this optimal distance.

Furthermore, we showed that our model performed better on predicting multiple TF binding sites than single TF binding sites, which fits well to the *cis*-module concept. This feature makes our model particularly appealing to predict cooperative TF binding sites. In the present study, the ChIP-chip data is only available to the 1% of human genome (ENCODE regions). With the availability of ChIP-seq data, we expect our model will become readily scaled up to the whole genome.

## Materials and Methods

### ChIP-Chip experiments using tiling array

Hela S3 cells from American Type Culture Collection (ATCC) were grown in DMEM supplemented with 10% fetal bovine serum, 2 mM glutamax and penicillin/streptomycin. Cells were crosslinked with 1% formaldehyde for 20 min at room temperature, washed with cold PBS three times and stored at −80°C. Antibodies against E2F1 (sc-193), E2F4 (sc-1082x), MAX(sc-197), YY1(Sc-7341) were obtained from Santa Cruz Biotechnology, CA. Antibody specific against phospho-Creb(5322) was a kind gift from Dr. Marc Montminy. Magnetic beads carrying sheep secondary antibodies were from Dynal (Invitrogen, CA). For chromatin Immunoprecipitation crosslinked cells were lysed and isolated nuclei were lysed and DNA was sheared in a Branson-450 sonifier for 15 cycles of 30 seconds each at 50% power with 120 seconds cooling on ice between each sonication. Primary antibodies immobilized on magnetic beads were used to immunoprecipitate the chromatin and were washed several times in RIPA buffer. DNA was then recovered from the beads following reverse-crosslinking and purification by proteinase K and RNAse A treatments. A small portion of the starting chromatin was also purified in a similar way.

The immunoprecipitated DNA along with 20 ng of input sample were amplified using a ligation-mediated PCR. Amplified input and IP samples were labeled using Cy3 and Cy5 labeled dCTPs respectively and hybridized together to a PCR microarray carrying 24,537 non-repetitive sequences that are greater than 100 bps within the 44 ENCODE region. Each Chip-Chip experiment was performed at least three times each from three independently grown batches of Hela S3 cells. The dataset is found at http://nash.ucsd.edu/CIS/.

### Training an HMM

The conventional HMM training algorithm treats the sequence equally and calculates the likelihood of the HMM parameters using the given sequences. It is often required to assign a path of the HMM states to a sequence. To guide a sequence path to the corresponding HMM states, a class HMM has been suggested [28]. A class HMM calculates the forward and the backward variables while restricting its training to a path where the label of a sequence matches to the label of a HMM state. It assigns only one label to a symbol of a sequence. However, we may need to assign a sequence with a set of labels. For example, a portion of a sequence can be a target of several TFs. If we assign a label to each TF, the sequence needs to be labeled with multiple symbols. To assign multiple labels to a given set of sequences, we employed the training method used for gene detection in *Drosophila* [29]. This method assigns a probability distribution over labels to each base in the sequence considering ChIP-chip ratio and sequence information. The training algorithm is modified so that each path of a sequence is weighted using the probability distribution assigned to the sequence.

Firstly, to assign labels to the sequences we searched for the binding site candidates using sequence and ChIP information. Using GITTAR [23] we calculated the probability of being a binding site of each TF given the background and ChIP-chip ratio. A set of labels are assigned in the next step on the putative binding sites. The probability of being a target is treated as a confidence weight [29] and used to normalize the label probabilities. If the confidence is close to 1, the feature is considered certain, whereas if it is close to 0, it is considered totally uncertain. Next, we assigned a probability distribution of the *n*+1 labels (number of TFs (*n*) and background) to the binding sites candidates. We used ChIP-chip ratio to assign the label probabilities. If a binding is a target of 2 Tfs, 3 labels (including background) are assigned. Table 3 lists the value assigned to each label based on the ChIP-chip ratio (before normalization). We assigned a probability of 1 to a putative TF region of a positive probe, 0 to a negative probe, and 0.5 to an obscure probe. The background label has an adjustable probability of *v_x_*. Changing the value of *v_x_* can change the ratio between the probability of being background and that of being TFBSs. We used pseudo counts (0.01) for non-binding regions. The label probabilities are normalized to have a sum of 1. For example, if the ChIP-chip ratio of a probe is positive for CREB and E2F1, negative for YY1, and obscure for MAX, the probabilities of the probe to be the binding site of each TF are set to: 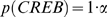, 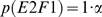, 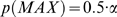, *p*(*YY1*) = 0, 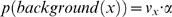, where 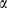 is a normalization factor. When *v_x_* is set to 0.5, 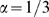, *p*(*CREB*) = *p*(*E2F1*) = 1/3, *p*(*MAX*) = 1/6, *p*(*YY1*) = 0, and *p*(*background*) = 1/6.

**Table 3:** Assigned value on each label based on the ChIP-chip ratio.

	Positive probe	Obscure	Negative probe	Background
Putative TFBS	1	0.5	0	*v_x_*
Non-binding regions	0.01	0.01	0	*v_x_*

The probability of being a target of a TF is used as a confidence of the label probabilities. If the probability is 1, we fully trust the label probabilities assigned using the ChIP ratio. If the probability is 0, the label probabilities are evenly assigned. The final label probabilities assigned to a sequence is calculated using
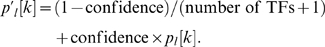
(2)



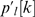
 is a weight multiplied to guide the calculation of likelihood to the legal path with given probabilities. The probability of yielding a sequence 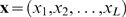 along a path 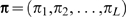 and a label 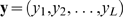 in an HMM is
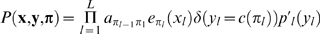
(3)where 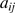 is the transition probability from state *i* to state *j*, and 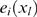 the probability of emitting a symbol 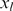 in state 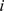. 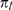 denotes the HMM state that the 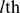 element of a sequence visits. 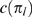 is the label in the state 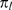. 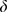 is the Kronecker delta function. It is 1 if 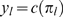, and 0, otherwise. These probabilities are multiplied along a path, so the probability of not using a path with high label probabilities is heavily penalized. Without the penalty term associated with labels (3) becomes
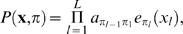
(4)which is the classical equation to calculate the likelihood of a sequence given an HMM [25]. The classical forward and backward algorithm is used to calculate the probability and the EM algorithm [25] is then utilized to update the emission and transition probabilities of the HMM. The modified training algorithm calculates forward and backward algorithm while considering label probabilities assigned to a sequence. An HMM's performance usually is affected by the initial parameters and its structure. As we chose our HMM with relatively simple structure, the initial parameters do not significantly affect the performance.

To find out bindings of a TF to a sequence we used the posterior label probability (PLP). The PLP calculates probability of a label of each TF to a sequence. The PLP of a label at a position is the sum of posterior probability of all states that emit the same label.
